# Nano-Assisted Radiotherapy Strategies: New Opportunities for Treatment of Non-Small Cell Lung Cancer

**DOI:** 10.34133/research.0429

**Published:** 2024-07-23

**Authors:** Lihong Zhao, Mei Li, Chen Shen, Yurui Luo, Xiaoming Hou, Yu Qi, Ziwei Huang, Wei Li, Lanyang Gao, Min Wu, Yao Luo

**Affiliations:** ^1^ West China Hospital, Sichuan University, Chengdu 610041, China.; ^2^ The Affiliated Hospital of Southwest Medical University, Southwest Medical University, Luzhou 646000, China.; ^3^ Zigong First People’s Hospital, Zigong 643000, China.

## Abstract

Lung cancer is the second most commonly diagnosed cancer and a leading cause of cancer-related death, with non-small cell lung cancer (NSCLC) being the most prevalent type. Over 70% of lung cancer patients require radiotherapy (RT), which operates through direct and indirect mechanisms to treat cancer. However, RT can damage healthy tissues and encounter radiological resistance, making it crucial to enhance its precision to optimize treatment outcomes, minimize side effects, and overcome radioresistance. Integrating nanotechnology into RT presents a promising method to increase its efficacy. This review explores various nano-assisted RT strategies aimed at achieving precision treatment. These include using nanomaterials as radiosensitizers, applying nanotechnology to modify the tumor microenvironment, and employing nano-based radioprotectors and radiation-treated cell products for indirect cancer RT. We also explore recent advancements in nano-assisted RT for NSCLC, such as biomimetic targeting that alters mesenchymal stromal cells, magnetic targeting strategies, and nanosensitization with high-atomic number nanomaterials. Finally, we address the existing challenges and future directions of precision RT using nanotechnology, highlighting its potential clinical applications.

## Introduction

Lung cancer has become the leading cause of cancer-related deaths and is the second most frequently diagnosed cancer. The number of deaths caused by lung cancer is almost 2.5 times higher than that of the second leading cause of cancer-related death, which is colorectal cancer [1]. Lung cancers are generally divided into 2 types: non-small cell lung cancer (NSCLC) and small cell lung cancer (SCLC). NSCLC is more common and includes various types of bronchogenic carcinomas, such as bronchi adenocarcinoma, squamous cell carcinoma, and large cell undifferentiated carcinoma [2].

Radiotherapy (RT) is a crucial component of cancer treatment. It is estimated that over 70% of lung cancer patients will need RT at some point during their disease progression [3]. In NSCLC, RT is the treatment modality indicated in almost all stages, importantly improving the local control or extending the overall survival of NSCLC patients [4–6]. RT primarily interacts with tumor cells through ionizing radiation (IR), which exerts a therapeutic effect and causes damage through direct and indirect actions. Directly, IR can impact biomolecules, particularly DNA. Indirectly, biomolecule damage is primarily caused by reactive oxygen species (ROS) [7–9]. Although some studies have implemented new technical measures to optimize RT, there are still limitations and side effects to consider. Tumor microenvironment (TME), characterized by abnormal tumor vasculature and a low oxygen microenvironment, can lead to radioresistance. Additionally, factors such as tumor type, size, location, distance, and patient characteristics pose challenges for RT planning. Furthermore, RT inevitably damages normal surrounding tissues, causing side effects such as skin cell damage, nausea, and vomiting. Increasing radiation dose to eliminate tumors can intensify side effects and accelerate tumor resistance development. Fortunately, advanced technologies have been applied to RT, extending its effectiveness while reducing potential hazards. For instance, image-guided RT helps locate and analyze tumor spread [10]. Other promising areas of research involve combining immunotherapy with RT [11], radionuclide-based treatment [12], concurrent chemoradiotherapy, and targeted therapy. Additionally, the treatment regimen for NSCLC often requires systemic therapy and a combination of multiple treatments. It is hoped that further advancements in technology will continue to enhance the efficacy of RT when used in combination with other treatments.

Nanomedicine refers to the application of nanotechnology in medicine and has made significant progress in the field of cancer treatment [13]. Nanotechnology involves the use of materials with sizes ranging from 1 to 100 nm, specifically designed for medical therapies and devices [14]. These materials offer advantages such as signal amplification, biocompatibility, easy modification, and excellent stability. In terms of medical utilization, nanomaterials can be categorized into 4 types: nanometallic biomaterials, inorganic nonmetallic biomaterials, polymer biomaterials, and composite biomaterials. In recent years, the understanding and exploration of nanomedicine have led to the increasing use of nanotherapeutics and nanotechnologies in clinical imaging, detection, diagnosis, RT, drug delivery, and nanobiosensors [15–18]. For instance, liposomes can be utilized to create nanodrug delivery systems. By consisting of a lipid bilayer and enclosed aqueous spaces, liposomes have been developed for encapsulating doxorubicin to enhance chemotherapy for lung cancer [19,20]. In terms of detection equipment, nanomagnetic beads have been employed for nucleic acid extraction. Moreover, nanotechnology can play a role in enhancing tumor accumulation and penetration, stabilizing drug release, and improving cellular drug absorption, thereby assisting in cancer therapy [21–25]. Notably, nanotechnology has made significant breakthroughs in the treatment of lung cancer, including the improvement of standard therapies such as surgery, RT, and chemotherapy. DNA nanorobots aid in surgical operations by targeting and preventing tumor development, while multiple nanomaterials help overcome drug resistance and enhance the effects of RT or drugs. Recently, the combination of nanotechnology and RT has emerged as a hot research field [26,27].

Nanotechnology shows great promise in precision RT [28]. In order to improve the effectiveness of RT for optimal tumor treatment outcomes, it is essential to first understand the specific mechanisms by which nanomaterials contribute to the therapeutic process. The first mechanism is nano-assisted radiosensitization, which involves the promotion of the accumulation of radiosensitizers at the tumor site through the enhanced permeability and retention effect (EPR) [29]. EPR benefits from the abnormal vascular morphology and structure of tumors, which include a lack of smooth muscle and large intercellular spaces. This allows nanoparticles (NPs) to effectively penetrate into the tumor. Additionally, the absence of lymphatic vessels and the obstruction of lymphatic return further enhance NP accumulation [30,31]. Nanomaterials can be coated with other biomolecules, such as albumin (Alb), to facilitate high accumulation in tumor sites and enable low-dose radiation to take effect. The second mechanism is the nano-assisted regulation of TME. In addition to the influence of nanomaterials and radiation, tumor cells and the TME are also important factors that cannot be ignored. Cancer-associated fibroblasts (CAFs), immune cells, extracellular matrix (ECM), and blood vessels in the TME all impact radiosensitivity. CAFs enhance the radiation tolerance of tumors by improving the DNA damage resistance of tumor cells and their ability to repair DNA after damage [32]. The ECM can promote the proliferation of tumor cells after RT damage by enriching tumor growth factors and cytokines, which assists in the radiation resistance of tumors. Immunosuppressive cells in the TME hinder the antitumor immune response following RT. The depletion of antitumor immune cells further impairs the antitumor function of the immune system [33]. Abnormal vascular structure in the TME is a significant cause of radioresistance. After RT, tumor cells secrete a large quantity of prosurvival cytokines, inhibit endothelial cell apoptosis, prevent vascular damage, and weaken the anticancer radiation effect [34]. Another crucial factor contributing to poor RT effectiveness is hypoxia. Because the reactivity of tumor cells to IR relies heavily on the presence of oxygen, hypoxia significantly increases the radiation resistance of tumors [35,36]. Studies have indicated that nanotechnology can help regulate the TME, thereby enhancing the body’s ability to kill tumors and synergistically improving the efficacy of RT. Nanomedicine also offers strategies to mitigate the side effects of RT on the body. Nanomaterials can function as carriers loaded with radioprotectors or can protect normal healthy tissue from damage based on their inherent properties. Therefore, it is crucial to carefully select the appropriate nanomaterial substrate, taking into consideration its physicochemical properties. Precise control over the size, shape, and surface characteristics of the nanomaterial is necessary to optimize its distribution in biological systems and enhance its sensitivity to radiation. Throughout the design process, it is important to consider the biocompatibility and safety of the nanomaterials to prevent excessive immune responses or toxicity in the host organism. Ultimately, in vitro assays and animal models are used to confirm the safety and radiosensitizing effects of the nanomaterials, providing a basis for their potential use in clinical settings [37].

In this review, we highlight the application of nanotechnology for enhanced RT. Nano-assisted RT strategies are introduced according to the mechanisms, including nanomaterials as radiosensitizers, nano-assisted regulation of the TME, nano-based radioprotectors in cancer, and radiation-treated cell products for indirect cancer RT. This review also discusses the current application of nano-assisted RT in NSCLC, which is divided into 3 parts: biomimetic targeting strategies based on a modification of mesenchymal stromal cells (MSCs), magnetic targeting strategies, and sensitization based on high-atomic number nanomaterials for enhancing RT of NSCLC.

## Current Status of RT for NSCLC

Depending on the size and location of the tumor, the degree of metastasis, and the difficulty of surgery, NSCLC is classified into 5 stages. RT is the treatment modality indicated in almost all stages of NSCLC, playing a significant role in initial treatment, adjuvant treatment, and posttreatment monitoring. For the locally early NSCLC (stages I and II), a surgical operation is the major treatment. When performing surgery, if the surgical margin is positive, the primary options are RT, chemotherapy, or chemoradiotherapy (simultaneous or sequential). However, in cases where the mediastinal nodes are negative and a patient is unable to undergo surgery, definitive RT and definitive chemoradiotherapy are the primary treatments available. Stereotactic body radiotherapy (SBRT), also known as stereotactic ablative radiotherapy (SABR), is a highly precise RT technology that delivers targeted high-dose radiation directly to tumors. This is made possible by advancements in tumor imaging, dosimetry, and the technology used to administer radiation. While SBRT and SABR are considered to be the same treatment technique, SABR is more accurate in indicating its therapeutic intent of tumor ablation. Clinical studies have shown that SABR not only directly destroys tumor cells but also triggers indirect antitumor effects, such as damaging the tumor’s blood vessels and activating the immune system. Furthermore, SABR is superior to conventional RT in terms of preserving normal tissue, requiring a shorter treatment time, and being more tolerable [4,38,39]. In cases of tumor recurrence after radical treatment, patients may opt for external irradiation radiotherapy (EBRT), brachytherapy, SABR, or concurrent chemoradiotherapy. For distant metastases such as local symptoms, diffuse brain metastases, bone metastases, etc., the main RT option is palliative external beam therapy [40]. In summary, RT plays a crucial role in the comprehensive treatment of NSCLC throughout the entire process.

However, current RT has certain limitations that need to be addressed. First, biological changes in the tumor and surrounding microenvironment, such as abnormal vascular distribution and hypoxic conditions, can make the tumor resistant to radiation. Second, the effect of RT is affected by tumor heterogeneity, including intertumoral heterogeneity, the same type of tumor results in tumor specificity due to patient differences, and intratumoral heterogeneity, heterogeneity arising from different cell populations of the same tumor [41]. Last, there are inevitable side effects. While RT kills tumor cells, it can also harm the surrounding healthy tissues. Additionally, the ability of the tumor adjacent tissues to tolerate radiation directly impacts the effectiveness of RT. Due to these limitations, it is necessary to combine RT with various treatment methods in order to enhance its impact on tumors and improve patient outcomes.

With the advent of precision medicine and advanced technologies for anticancer treatment, numerous studies have been conducted to enhance the treatment of NSCLC. RT plays a crucial role in the management of NSCLC, and it is essential to improve its precision. RT can be classified into radical, adjuvant, and palliative therapy, depending on the treatment goals. In the comprehensive treatment approach for NSCLC, RT typically serves as an adjunct and radical therapy. Timmerman et al*.* conducted a trial to assess the adverse reactions and therapeutic efficacy of SBRT in patients with inoperable early-stage lung cancer. SBRT employs the use of stereotactic techniques and specialized radiation equipment to precisely target the tumor area [42]. It offers the advantage of delivering a high radiation dose in just one treatment, requiring fewer sessions overall, while also providing maximum protection to the healthy tissues surrounding the tumor, minimizing potential damage. In comparison to conventional treatment, which has a low success rate in controlling tumors and a high mortality rate, with a 3-year survival rate below 35%, SBRT significantly improves these outcomes, with over 50% of patients surviving at the 3-year mark [43]. Moreover, a general population-based study has demonstrated that SBRT can improve the overall survival of elderly patients with stage I NSCLC [44]. SBRT may improve the local control in unresectable stage III NSCLC under favorable conditions [45].

During the therapeutic process of NSCLC, chemoradiotherapy is a common treatment modality. These options include concurrent radio-chemotherapy and sequential chemoradiotherapy. For example, the National Comprehensive Cancer Network (NCCN) guideline recommends the use of paclitaxel and carboplatin with concurrent thoracic RT, as well as cisplatin plus etoposide with concurrent thoracic RT, for squamous cell carcinoma [46]. Chemoradiotherapy is considered superior to RT alone for locally advanced NSCLC in patients with unresectable stage IIIA or stage IIIB disease. Concurrent radio-chemotherapy is found to be more effective than sequential chemoradiotherapy. However, concurrent chemoradiotherapy has been linked to a higher incidence of grade 3 or 4 esophagitis when compared to sequential chemoradiotherapy [47].

Furthermore, in terms of the future direction of RT, the combination with immunotherapy is gaining significant attention in the research field. The discovery of programmed death receptor 1 (PD-1) and programmed death ligand 1 (PD-L1), as well as the emergence of immune checkpoint inhibitors, has brought about significant advancements in the treatment of advanced lung cancer. By performing PD-L1 testing, it is now possible to identify patients who are more likely to benefit from immunotherapy. This information can guide subsequent decisions regarding chemoradiotherapy or systemic treatment [48]. Immunotherapy has demonstrated promising clinical efficacy in patients with NSCLC. Pembrolizumab, when combined with platinum chemotherapy, has proven to be an effective first-line immune checkpoint inhibitor for late-stage NSCLC patients [49]. A recent randomized controlled trial, utilizing RNA-sequencing and immunohistochemical analysis data from NSCLC tumor samples, has provided evidence of the antitumor effect of combining immunotherapy with chemotherapy in advanced NSCLC patients with high PD-L1 expression [50]. However, the response to these immunotherapies is highly reliant on the specific tumor immune microenvironment, as indicated by the expression of various biomarkers such as PD-L1, tumor-infiltrating lymphocytes (TILs), and tumor mutational burden (TMB). Consequently, not all late-stage NSCLC patients can benefit from immune therapy, which is a significant limitation in the development of immunotherapy [51]. RT can affect the antitumor immune responses by boosting the release and delivery of antigens and remodeling the TME to improve the therapeutic effect of immunotherapy [52–54]. SBRT has powerful immune activation, and PD-1/PD-L1 inhibitors can attenuate radiation resistance. A combination of SBRT and PD-1/PD-L1 inhibitors can improve antitumor immunity [55]. A study has revealed that with the use of RT, oxidative phosphorylation (OXPHOS) is up-regulated, not only helping cancer cells adapt to this change but also increasing tumor sensitivity to OXPHOS inhibitors [56,57]. RT combined with a novel OXPHOS inhibitor, which affects the immune therapeutic effect, can be an innovative way to deal with PD-1-resistant NSCLC [58]. However, only a limited number of clinical trials have demonstrated the therapeutic effect of combining RT with immunotherapy for NSCLC. Trials investigating the toxicity and the impact of RT on the host immune system have indicated that higher radiation doses may result in immunotoxicity, which can be a significant factor contributing to tumor progression [59–62].

**Fig. 1.**
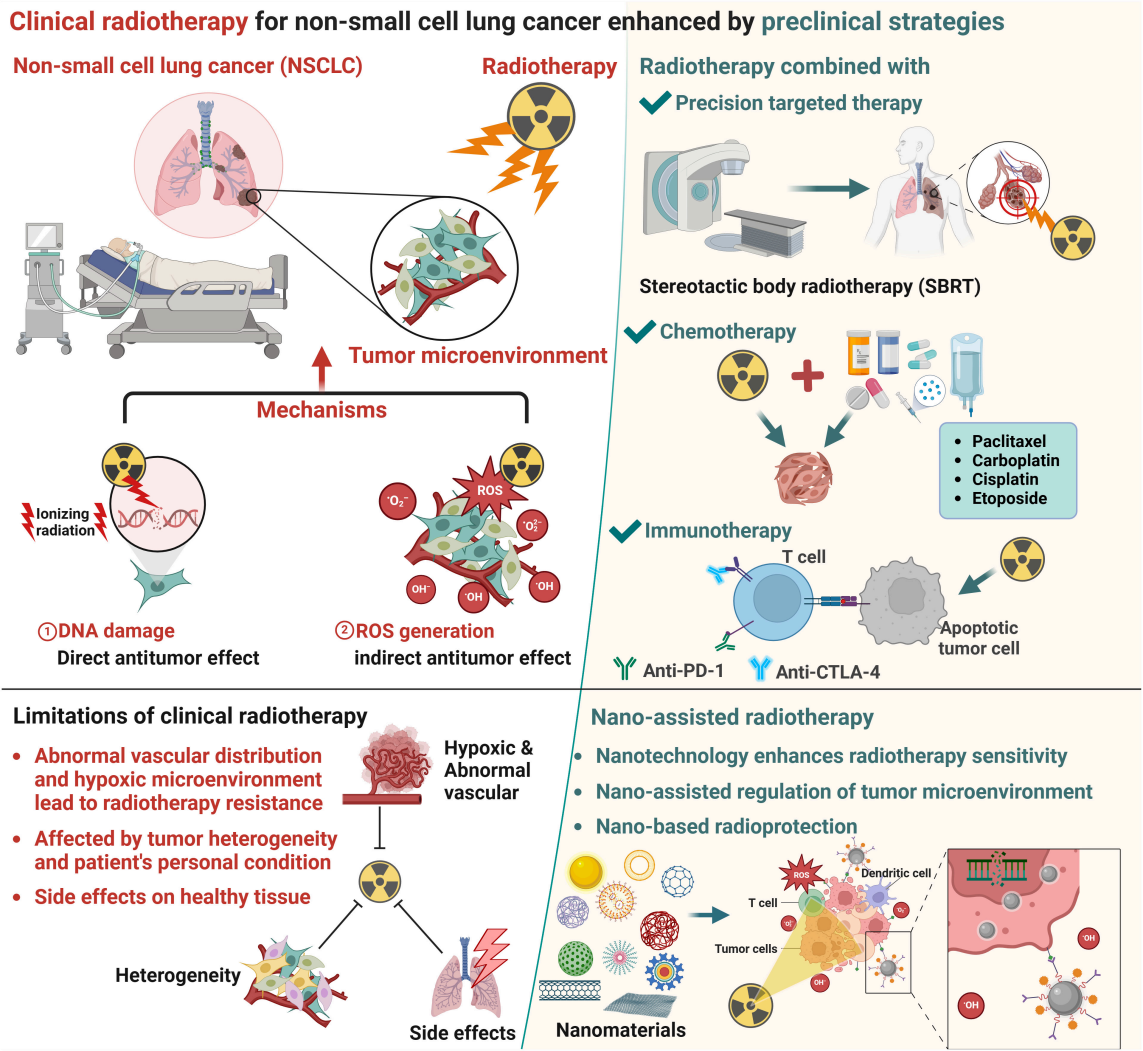
Clinical radiotherapy for NSCLC enhanced by preclinical strategies. RT exerts antitumor effects through DNA damage and ROS production. Abnormal tumor vascular distribution and hypoxic TME induce tumor cell resistance to RT. Tumor heterogeneity and patients’ complex health status also affect the efficacy. In addition, RT often has unavoidable side effects, increasing the burden on patients. The application of several new technologies has improved the status quo of RT for NSCLC, including targeted therapy, chemotherapy, and immunotherapy. The application of nanomaterials can increase the sensitivity of NSCLC to RT, exert antitumor effects by regulating the TME, and provide radioprotection.

Overall, the strategies above all have a common goal of improving the outcome of anticancer therapies and patients’ lives. It is necessary to optimize the RT for NSCLC along with advancing preclinical technologies. Therefore, the combination of RT and immunotherapy for NSCLC needs to be rigorously validated to ensure the positive effects of this treatment mode and avoid negative effects. In recent years, it is worth noting that nanotechnology can be a viable strategy to help optimize RT and improve the therapeutic outcome of NSCLC patients (Fig. 1) [63–68].

## Mechanisms of Nano-Assisted RT in Cancer

**Fig. 2.**
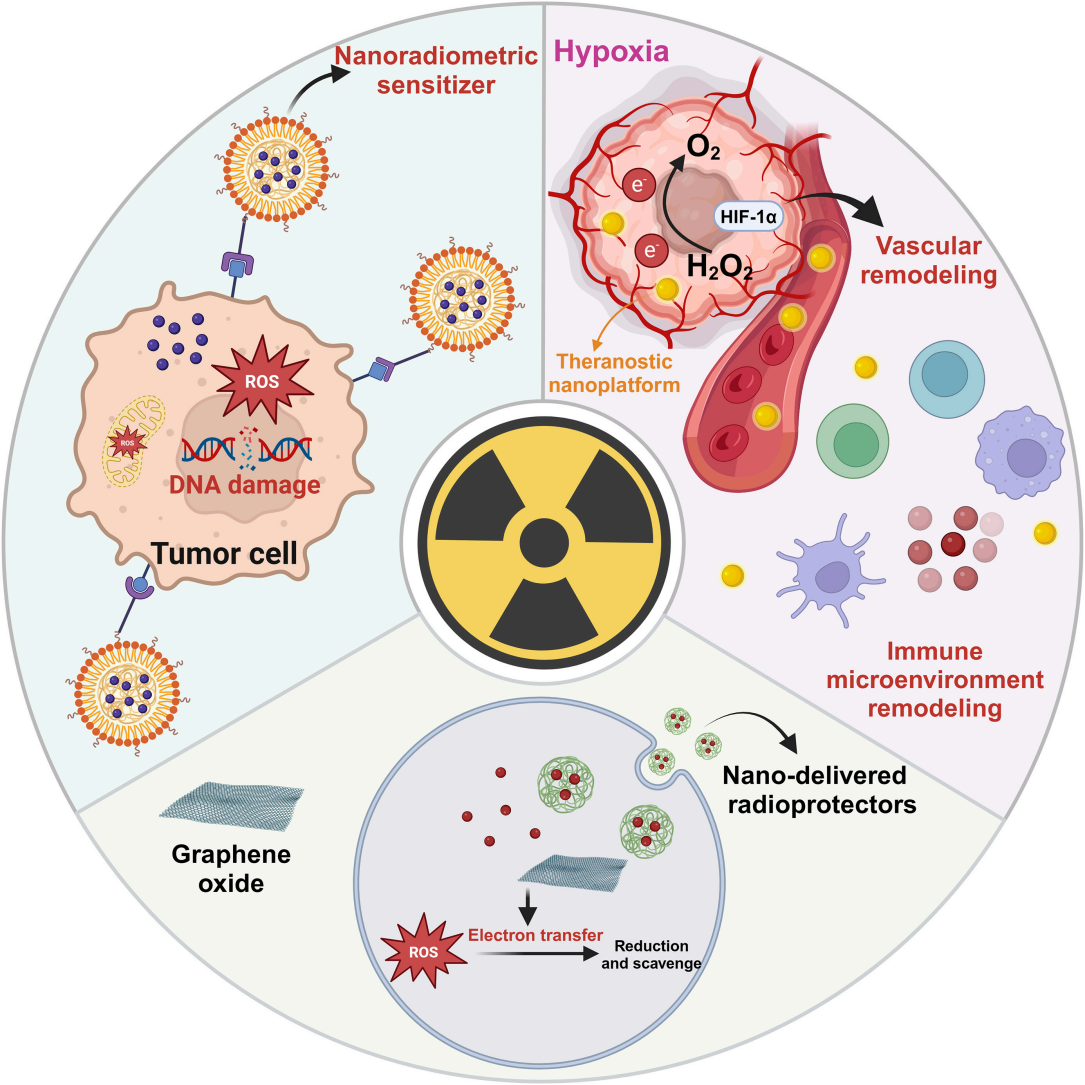
Mechanisms of nano-assisted RT in lung cancer. Nanotechnology enhances the sensitivity of RT by generating ROS and oxidative stress. Additionally, nanotechnology can modulate the TME by alleviating hypoxia, overcoming vascular abnormalities, and reshaping immune microenvironment. Furthermore, nanotechnology aids in reducing the side effects of RT by facilitating the delivery of radiation protectants and scavenging free radicals.

When tumor cells are exposed to radiation, RT can kill them in 2 main ways. The primary method is by destroying biological molecules, such as DNA, which leads to disruptions in cell growth, apoptosis, and necrosis. Another method involves ROS damage to biomolecules, which is also crucial for tumor cell apoptosis. The use of nanoradiosensitizers improves the therapeutic effect of RT by leveraging these 2 mechanisms. Additionally, TME also impacts the outcome of RT. Numerous nanomaterials are specifically designed to enhance the efficiency of RT by precisely targeting tumors, increasing radiation energy absorption and damage in tumor cells, and regulating the tumor environment. Furthermore, the application of nanotechnology can reduce side effects and protect normal tissues (Fig. 2). Finally, we discuss the latest studies on the use of radiation-treated cell products for indirect cancer RT.

### Nanotechnology enhances RT sensitivity

**Fig. 3.**
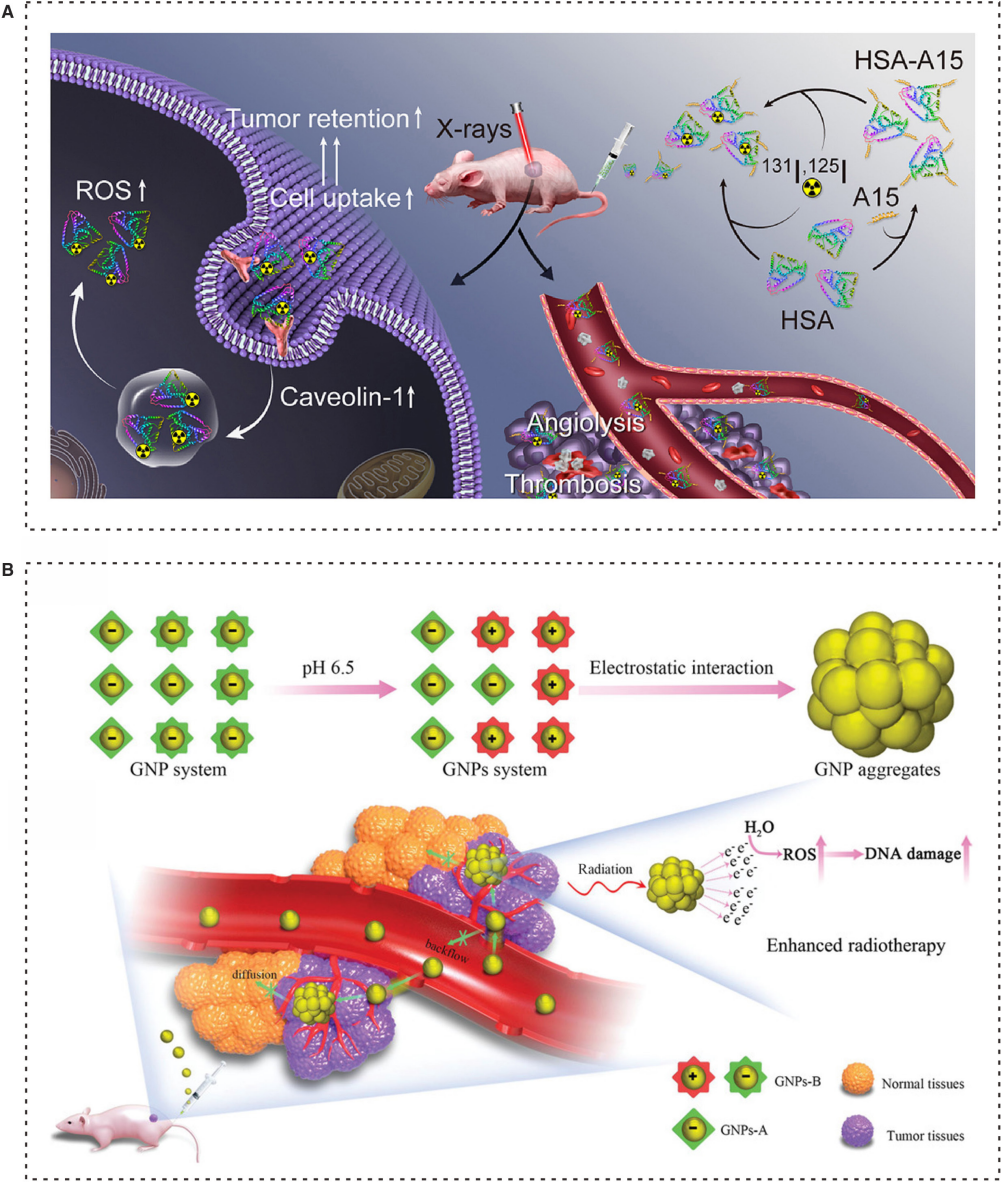
Nanotechnology enhances RT sensitivity. (A) X-ray irradiation increases expression of caveolin-1 and enhances RT sensitivity by promoting cellular uptake of HSA NPs and prolonging tumor retention. Adapted with permissions from [75]. Copyright 2020, Elsevier. (B) Acid-triggered small-size GNPs accumulate within tumors and act as tumor radiosensitizers by generating more ROS to damage DNA. Adapted with permissions from [87]. Copyright 2019, John Wiley and Sons.

Nanotechnology has the potential to enhance the sensitivity of tumor RT through various mechanisms. On the one hand, enhancing radiosensitivity is primarily achieved through the generation of ROS and oxidative stress. Additionally, when NPs are combined with other materials through synergistic mechanisms, the reactive process is enhanced, leading to an amplified radiobiological response. On the other hand, the biological mechanism involves damaging DNA, as well as other structures and molecules that can be targeted for radiosensitization, such as mitochondria and cell membranes. Furthermore, the presence of nanomaterials also influences the biosystem in the body, which can impact the regulation of the cell’s biological cycle and accelerate the process of apoptosis. Therefore, it is important to consider potential toxicities associated with these effects as well [69]. In summary, achieving accurate and effective RT with nano-assisted radiosensitization requires closely integrating experimental and clinical practice and considering many factors (Fig. 3).

DNA damage is commonly considered a primary marker for evaluating the effects of radiosensitizers. While studies have confirmed that NPs can enhance cell killing when combined with radiation, this increase is not solely attributed to DNA damage. Other factors, such as mitochondrial dysfunction and cell membrane damage, also contribute to the killing of cancer cells [70,71]. Recently, researchers have been investigating the use of radiosensitizers that target specific organelles such as mitochondria, endoplasmic reticulum, and lysosomes. Tang et al*.* utilized triphenylphosphonium (TPP), an organic compound known for its selective targeting of mitochondria, to modify Gd-doped titanium dioxide NPs [TiO_2_(Gd) NPs]. By introducing TPP, the resulting NPs were named TiO_2_(Gd)-TPP NPs. Subsequent experiments demonstrated the specific targeting ability of TiO_2_(Gd)-TPP NPs toward mitochondria and their ability to generate ROS, which accelerated mitochondrial dysfunction and led to cell apoptosis. It is possible that radiosensitizers could target the cytomembrane, organelles, and molecular structure within a cell [72].

EBRT is the most widely utilized type of radiation oncology treatment. It involves the administration of high-dose radiation, such as x-rays, electron beams, or heavy ion beams, to either eliminate cancer cells or reduce the size of tumors. X-rays have the ability to harm DNA, infiltrate cells, and enhance the effectiveness of radiosensitizers by increasing their absorption [73]. Yi et al*.* have confirmed that the expression of caveolin-1 induces the cellular uptake of human serum Alb (HSA)-based NPs under x-ray irradiation. Additionally, x-ray irradiation can enhance the expression of caveolin-1. The higher the expression of caveolin-1, the more ROS is generated, which enhances the sensitivity of RT. HSA is commonly used as a nanocarrier for delivering drugs in tumor therapy. HSA-based NPs have the advantage of excellent tumor accumulation and long-term retention in the tumor due to their inherent biocompatibility [74]. They also demonstrated that x-ray irradiation can enhance the functionality of HSA-based NPs by modifying them with the A15 peptide [75].

The use of metal-based NPs for radiosensitization has been thoroughly investigated, including silver (Ag), gadolinium (Gd), and tantalum (Ta) [76–80]. Among these, gold nanoparticles (GNPs) are the most commonly used in nanotechnology-based neoadjuvant RT [81]. Recent studies have focused on maximizing the synthesis ability of GNPs to create multifunctional nanoplatforms and enhance their potential in clinical cancer RT [9,82]. Although GNPs have high x-ray energy that accumulates in tumor sites, their tumor-targeting ability is poor. Through the combination and modification of metal NPs, they work together to enhance radiosensitivity and promote better results [28]. For instance, Chen et al*.* developed a new nanoplatform called Alb-modified gold nanoparticles (Alb-GNPs). GNPs have the ability to enhance the absorption and accumulation of the effective radiation dose in tumor cells. They also increase the production of free radicals, which can damage DNA. Alb, serving as the carrier for the drug, improves the active targeting of tumors due to the presence of the Alb receptor and secretory protein (SPARC) that are highly expressed in tumors. This ensures the biocompatibility of GNPs. The combination of these 2 factors allows Alb-GNPs to achieve precise RT and remain in the bloodstream for a longer period of time [83,84]. Based on the acidic TME of tumor cells, Zhang et al*.* creatively designed a strategy for acid-triggered aggregation of small-sized GNPs. Small-sized GNPs could better solve the problem of leakage and diffusion from tumor cells. They used 2 kinds of peptides to modify GNPs into GNPs-A and GNPs-B; when GNPs-B reached the tumor site, in the acidic environment of the tumor, the negative charges on its surface were neutralized [85]. Then, GNPs-B and GNPs-A combined through electrostatic interaction, resulting in the formation of small-sized aggregates of GNPs. These aggregates have the ability to generate ROS when exposed to radiation and can accumulate at the tumor site for an extended period of time. The system could even achieve the goal of reducing toxicity to the body by clearing quickly [86,87].

### Nano-assisted regulation of the TME

**Fig. 4.**
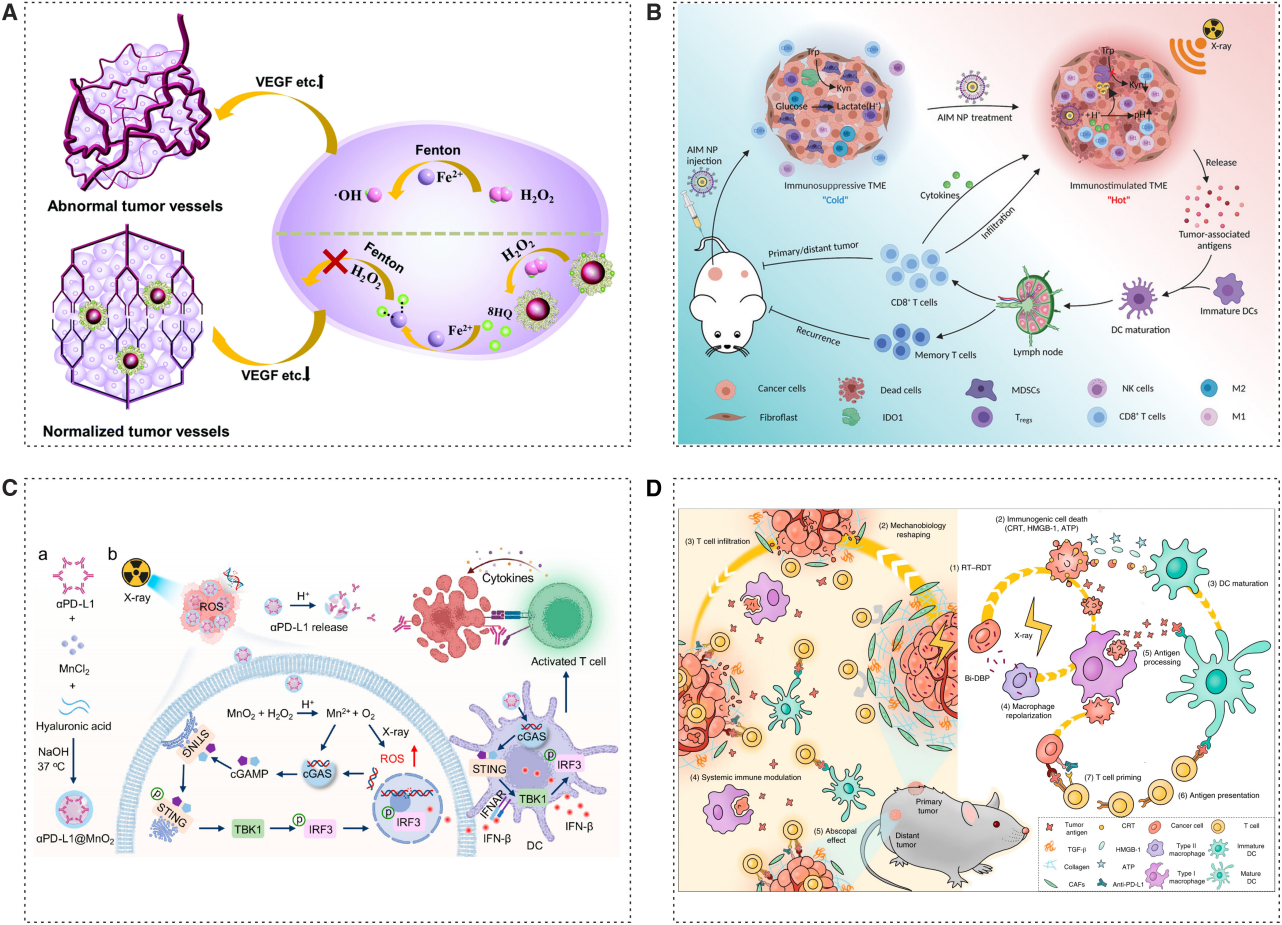
Nano-assisted regulation of the tumor microenvironment. (A) Au@SA-QBA synthesizes and normalizes tumor vasculature. SA-QBA can selectively react with H_2_O_2_ to produce phenols and play a strong coordination effect on iron, thereby inhibiting oxidative stress to help normalize tumor vasculature. Adapted with permissions from [102]. Copyright 2021, Royal Society of Chemistry. (B) AIM NPs can reverse immunosuppressive metabolic TME for reinforced RT. Adapted with permissions from [106]. Copyright 2022, John Wiley and Sons. (C) Schematic illustration to show the preparation of αPD-L1@MnO_2_ and its mediated delivery of checkpoint inhibitors to reinforce RT-induced systemic antitumor immune responses. Adapted with permissions from [109]. Copyright 2023, American Chemical Society. (D) Scheme of Bi- 5,15-di(*p*-benzoato)-porphyrin (DBP)-mediated RT-radiodynamic therapy (RDT) modulated biomechanics to promote T cell infiltration. Adapted with permissions from [110]. Copyright 2022, Springer Nature.

The microenvironment is another vital factor that influences the effectiveness of RT. Numerous studies have examined the metabolism of the TME and its influence on tumor progression. These studies have investigated the idea of metabolic reprogramming and how it affects tumor development and growth [88]. The TME is a complex environment that includes tumor metabolites, blood vessels, the ECM, CAFs, and immune-related cells and factors (secreted factors) [89–91]. RT can induce immunogenic cell death (ICD), a unique type of cell death that activates the immune system to fight against tumors. However, it also leads to radioresistance by creating an immunosuppressive tumor microenvironment (ITM). This environment is characterized by the presence of immune-related cells and regulators that suppress the immune response, including tumor-associated macrophages (TAMs) with the M2 phenotype, myeloid-derived suppressor cells (MDSCs), regulatory T cells (T_reg_ cells), and cytokines such as interleukin-10 (IL-10) and transforming growth factor-β (TGF-β). The body’s immune system seeks to achieve and maintain a state of balance. Additionally, tumor blood vessels differ from normal blood vessels, as they are distorted, irregular in shape, and highly permeable. This makes it challenging for chemotherapy drugs to effectively reach the tumor. Furthermore, the abnormal blood vessels limit the effectiveness of RT by contributing to the formation of a hypoxic TME [92]. Importantly, RT and TME mutually influence each other. The hypoxic TME limits the damage caused by RT. When radiation mediates tumor endothelial vessel injury, it recruits immune cells and activates the hypoxia-inducible factor-1α (HIF-1α)-induced pathway, leading to vascular remodeling [93,94]. This pathway also protects cells from radiation by influencing cellular metabolism and promotes the production of antioxidants [95]. Under the condition of radiation exposure, TME has changed in many aspects, such as blood vessels, stroma, increased hypoxia state, and immunity, which are also correlated with radioresistance and tumor recurrence [96]. NPs are used for remodeling TME and sensitizing RT, including 3 main mechanisms: alleviating hypoxia, overcoming tumor vascular microenvironment abnormalities, and rebuilding the ITM (Fig. 4) [97–99].

Normalizing the abnormal tumor vascular microenvironment is a significant strategy for RT enhancement. Compared with the normal cell, the multiplication of tumor cells needs more provision of blood, thus causing them to create new blood flow pathways. As a result, compression of surrounding tissues by blood vessels exacerbates the lack of oxygen, and because of the concentrated distribution of abnormal vascular, nanomaterials have difficulty playing their role in blood circulation [93]. A lot of strategies have been explored in normalizing abnormal tumor vascular microenvironments [100]. SA-QBA was formed by the reaction of sodium alginate (SA) and 8-quinoline boric acid (QBA), which can react with H_2_O_2_ to form a phenolic substance. The phenolic substance has been shown to avoid binding to metal ions in normal tissues and selectively binds to iron ions in tumor tissues to inhibit oxidative stress and angiogenic factors [101]. It had been designed to modify GNPs as Au@SA-QBA to normalize the abnormal tumor vascular microenvironment. As the experimental result shows, the hypoxic positive area was largely decreased by using Au@SA-QBA in contrast to the phosphate-buffered saline (PBS)-treated group [102]. Although hypoxia inhibits the sensitivity of RT, some studies have utilized this property to design hypoxia-responsive therapeutic drugs and radiosensitizers, which exert synergistic anticancer effects by regulating oxidative stress inside tumor cells [103]. In addition, the low pH characteristics of certain tumor TMEs can be utilized to achieve controlled and intelligent release of nanomedicines. Furthermore, this strategy can help reverse the treatment-resistant microenvironment by consuming acidic substances in the environment and alleviating hypoxia, thus increasing the tumor’s sensitivity to RT [104].

Recently, there has been a growing research focus on the use of nanotechnology to aid in the reconstruction of the ITM in the field of RT. Wang et al*.* designed a pH-responsive nanoplatform utilizing 4-phenylimidazole (4PI)-coated calcium carbonate (CaCO_3_) NPs complexed with zinc ions, termed as acidity-IDO1 modulation nanoparticles (AIM NPs). The indoleamine 2,3-dioxygenase 1 (IDO1) inhibitor 4PI can suppress the IDO1-mediated production of kynurenine (Kyn) upon tumor accumulation, while CaCO_3_ neutralizes tumor acidity by reacting with protons within the TME [105]. This platform primarily modulates the TME in 2 aspects to reduce RT resistance and enhance RT efficacy. First, leveraging the material’s properties, 4PI is released under tumor acidic conditions to inhibit IDO1-mediated Kyn production, thereby reversing acidosis-induced radioresistance and suppressing the generation of immunosuppressive Kyn. Second, the material promotes the infiltration and response of tumoricidal cells while concurrently inhibiting the frequency of immunosuppressive cells within the TME. By modulating the pH and immunosuppressive metabolism of the TME, this approach successfully inhibited tumor growth and augmented the effectiveness of RT [106]. The process of dendritic cell (DC) maturation can involve various factors, such as changes in the nearby microenvironment, the release of immunostimulatory molecules, and activation through pattern recognition receptors (PRRs) [107]. By modulating the pH of the TME and inhibiting the activity of IDO1, AIM NPs can provide a more conducive environment for the maturation of DCs. Notably, Deng and colleagues have synthesized a biomineralized manganese dioxide (MnO_2_)-based nanoplatform with high encapsulation efficiency of anti-programmed death ligand 1 (αPD-L1), denoted as αPD-L1@MnO_2_. This nanoplatform facilitates the release of Mn^2+^ ions stimulated by the acidic TME, which in turn activates and amplifies the cGAS–STING pathway to promote the maturation of DCs [108]. Consequently, this enhances the tumor-specific immune response following RT-induced ICD. Additionally, the released αPD-L1 can specifically block the interaction between PD-L1 on tumor cells and PD-1 on cytotoxic T lymphocytes (CTLs), further promoting the infiltration of CTLs within the tumor. Moreover, this platform is capable of catalytically generating oxygen, providing additional assistance for RT by alleviating tumor hypoxia [109]. Moreover, Ni et al*.* have synthesized and evaluated a bismuth (Bi)-based nanoscale metal-organic framework (nMOF) with the aim of modulating the immunological and mechanical properties of the TME. This novel platform, in addition to its capabilities of generating reactive species, facilitating ICD, and diminishing the levels of TGF-β within the intratumoral milieu, distinctively emphasizes the reduction of tumor rigidity conferred by Bi-nMOF treatment. This reduction in stiffness is instrumental in alleviating the physical barriers to T cell infiltration, a feature that sets it apart from previous studies [110].

Metal-based nanomaterials have certain limitations due to unresolved concerns regarding long-term in vivo safety and biodegradability. Zhang et al*.* have developed a hydrogel based on Toll-like receptor 7/8 (TLR7/8) agonists and a radiosensitive peptide (Smac-TLR7/8 hydrogel) [111,112]. This novel biomaterial is designed to modulate the polarization of TAMs, overcoming tumor resistance to RT. The advantages of this material over metallic counterparts include its ability to self-assemble into nanofibrous structures, which enhances the retention and bioavailability of the therapeutic agent in the body. Additionally, it ensures biocompatibility and minimizes side effects. The mechanisms involved in regulating the ITM and augmenting the efficacy of RT include macrophage polarization. The Smac-TLR7/8 hydrogel promotes a shift in macrophage polarization, stimulating an inflammatory response and enhancing antitumor activities. By binding to inhibitors of apoptosis proteins (IAPs) and enhancing radiosensitivity, the Smac-TLR7/8 hydrogel increases the sensitivity of tumor cells to RT, thus overcoming resistance to this treatment. Therefore, the Smac-TLR7/8 hydrogel facilitates the reconstruction of the ITM, reducing resistance to RT and improving the therapeutic outcomes of this treatment [113].

### Nano-based radioprotection

**Fig. 5.**
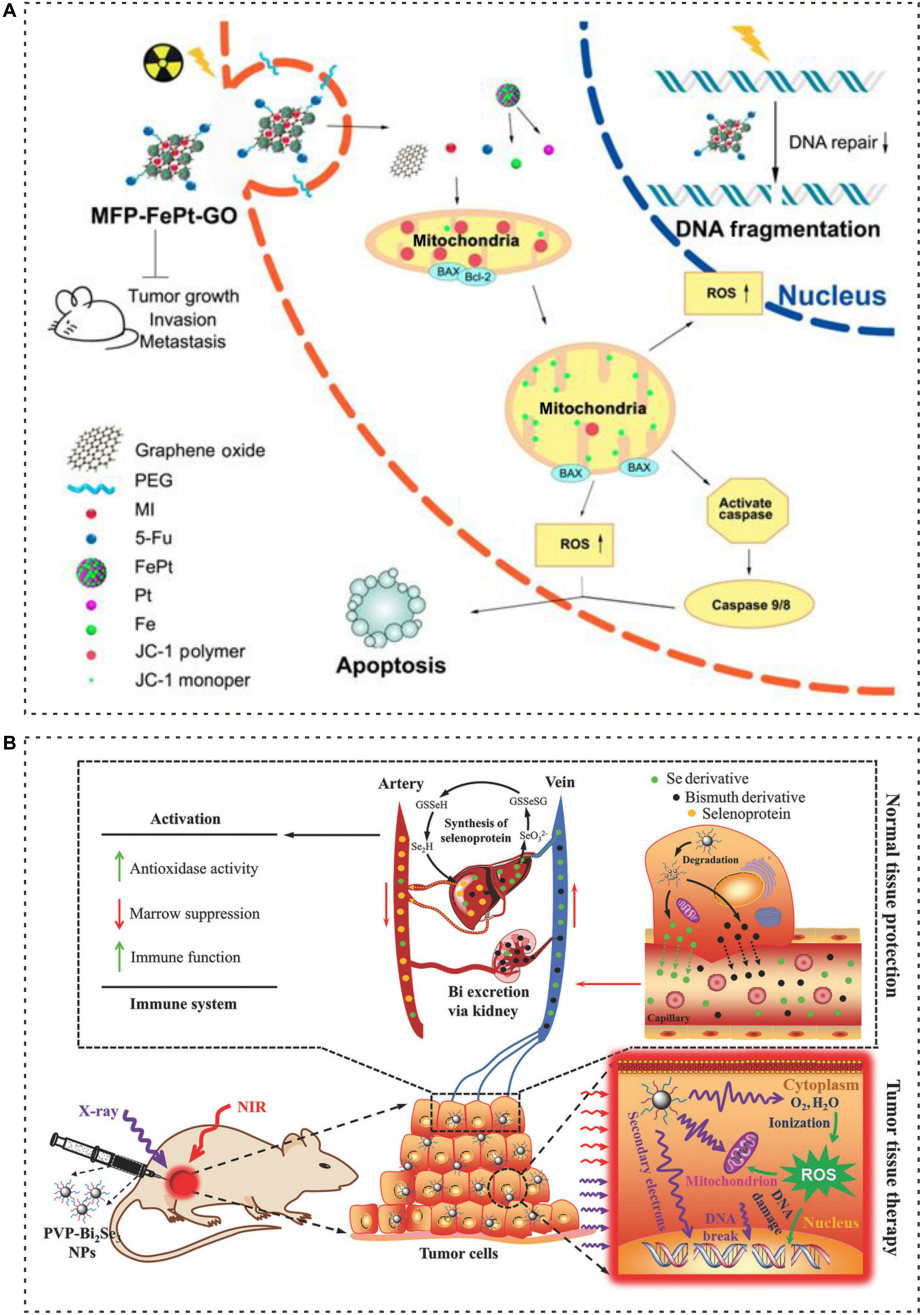
Nano-based radioprotection. (A) MFP-FePt-GO NCs induce DNA damage in tumor cells by changing mitochondrial membrane potential and up-regulating ROS levels under endocytosis, thereby increasing RT accuracy and reducing damage in healthy tissues. Adapted with permissions from [117]. Copyright 2020, Ivyspring International Publisher. (B) PVP-Bi_2_Se_3_@Sec NPs can simultaneously enhance the effectiveness of RT and reduce radiation side effects. In the body, part of selenium can be released from NPs, enter the blood circulation system, enhance immune function, reduce the side effects of radiation on the whole body, and especially reduce the bone marrow DNA inhibition. Adapted with permissions from [119]. Copyright 2017, John Wiley and Sons.

While RT effectively kills tumors, it can also have adverse effects on surrounding tissues. However, the use of multifunctional nanomaterials can help minimize this damage and protect the normal tissues nearby. As mentioned earlier, ROS is an indirect pathway through which RT kills tumor cells, but it also harms the normal tissues. Nanomaterials offer protective mechanisms for these surrounding tissues, such as delivering radiation protection agents and scavenging free radicals. These protective capabilities have been discussed in previous research on nanomaterials and radiation protection [114]. Several nanomaterials have been demonstrated to have the ability to scavenge ROS and have been used in combination with other drugs or materials for synthesis. Among these radioprotective nanomaterials, graphene-based nanomaterials have been proven to possess the capacity to scavenge free radicals (Fig. 5) [115,116].

In pursuit of the goal of radiation protection, researchers have developed graphene-coated metal nanohybrids with super electrocatalytic activity. These nanohybrids can remove free radicals through an electron transfer mechanism, effectively depleting the excessive ROS produced by IR. Ma et al*.* proposed the use of FePt NPs assembled on the surface of graphene oxide (GO), while Peng et al*.* reported the development of MFP-FePt-GO nanocomposites (NCs). Both of these studies demonstrated the radiosensitizing effect of the nanohybrids without causing significant cytotoxicity to normal lung cells [117,118]. Additionally, Du et al. utilized the mild solution method to develop a new type of Bi_2_Se_3_ NPs, which can enhance tumor RT sensitivity while also showing excellent biological safety and minimizing adverse reactions through various mechanisms. Bi_2_Se_3_ NPs have good near-infrared (NIR) absorption and x-ray attenuation ability, so they can generate ROS under x-ray irradiation against tumors. The special coating ensures that the NPs have good water solubility and biocompatibility. At the same time, in situ tumor injection mode of drug delivery can guarantee a high drug concentration locally, reducing systemic toxicity of treatment. More importantly, due to the instability of nanomaterials and photothermal effect, the controlled release of selenium into the bloodstream enhances the body’s immunity and mitigates the side effects of radiation, including bone marrow suppression. Bi_2_Se_3_ NPs not only enhance the radiosensitivity of tumors but also improve the radioresistance of healthy tissues [119].

### Radiation-treated cell products for indirect cancer RT

In tumor RT, the phenomenon where radiation causes oxidative stress and DNA damage in nearby unirradiated cells or tissues is known as the radiation-induced bystander effect (RIBE). Recent studies have shown that certain components of irradiated tumor cells, such as cell membranes, cell secretions, and microparticles (RT-MPs) released by these cells, possess significant antitumor capabilities and may play a crucial role in RIBE [120,121]. Wan et al*.* discovered that RT-MPs have the ability to kill tumor cells and reprogram the immune microenvironment. When RT-MPs are injected externally, they effectively induce ferroptosis in tumor cells and trigger ICD, which in turn activates the host immune response. Additionally, when TAMs engulf RT-MPs, they undergo a polarization from M2 type to M1 type, thereby reactivating their antitumor function. An interesting observation is that TAMs treated with RT-MPs express higher levels of PD-L1, which enhances the sensitivity of tumors to immunotherapy and demonstrates the potential of combining RT and immunotherapy. The induction of a broad immune response by RT-MPs, along with their promotion of RIBE, holds great significance in combating drug-resistant tumors and inhibiting tumor metastasis and recurrence [122,123]. Analysis using electron microscopy has revealed the vesicle structure of RT-MPs at the nanoscale, further showcasing their potential as a form of nanomedicine and for use in nanodrug delivery systems. Deng et al*.* utilized the potential of RT-MPs to induce ferroptosis and act as biocompatible carriers to develop a tumor nanodrug delivery system. To enhance the antitumor effect, they loaded the ferroptosis inducer RSL-3 into RT-MPs, which activated the cGAS–STING pathway and promoted the polarization of TAMs from M2 to M1. Additionally, they incorporated the mitochondria-targeting peptide CT20p into RT-MPs, leading to increased production of ROS and lipid hydrogen peroxide. This alteration changed the mitochondrial membrane potential, intensified ferroptosis, and synergistically exerted antitumor effects [124]. To further enhance targeting and regulation of the TME, Lu et al*.* genetically engineered RT-MPs and applied them to combat brain metastases (BRM) of lung cancer. They transfected tumor cells with lentiviral vector plasmids to enable RT-MPs to express a scavenging receptor B1 (SR-B1) targeting peptide. SR-B1 is expressed on the surface of blood–brain barrier endothelial cells, microglia, and Lewis lung cancer (LLC) cells. As a result, it effectively improves blood–brain barrier permeability and targets BRM cells and tumor-related M2 microglia. The nanomedicine also carries a ubiquitin-specific protease 7 (USP7) inhibitor, which activates the mitogen-activated protein kinase (MAPK) signaling pathway, effectively reprograms M2 microglia, improves ITM, and works in conjunction with RT-MPs to directly and indirectly inhibit BRM [125,126]. In addition to nanodrug delivery systems, irradiated tumor cell products are also used in the construction of tumor vaccines. Tuo et al*.* utilized irradiated tumor cell membranes to wrap polylactic-glycolic acid (PLGA) NPs, thereby creating a tumor vaccine that enhances host immunity. This particular type of irradiated tumor cell membrane expresses a higher level of major histocompatibility complex I (MHC-I) and contains more damage-associated molecular patterns (DAMPs), resulting in stronger immunogenicity. The biomimetic NP is loaded with a TLR7 agonist (R837), which effectively accumulates in lymph nodes and activates DCs, thereby inducing antitumor immune responses [127,128]. In summary, the use of irradiated tumor cell components to prevent or treat tumors by simulating the occurrence of RIBE is a promising strategy for indirect RT. This approach demonstrates effective tumor eradication and immune activation capabilities.

## Strategies for Nano-Assisted RT in NSCLC

The current study in clinical settings focuses on the adjuvant role of various nanomaterials in the RT treatment of NSCLC. In this section, we will present an overview of the most recent research discoveries related to nano-assisted RT for NSCLC. This will involve delving into biomimetic targeting strategies that involve modifying MSCs, investigating magnetic targeting methods, exploring a drug-loaded magnetic materials system that operates under a magnetic field (MF), and investigating nanosensitization techniques that rely on high-atomic number nanomaterials [129,130].

### Biomimetic targeting strategies based on the modification of MSCs for RT of NSCLC

**Fig. 6.**
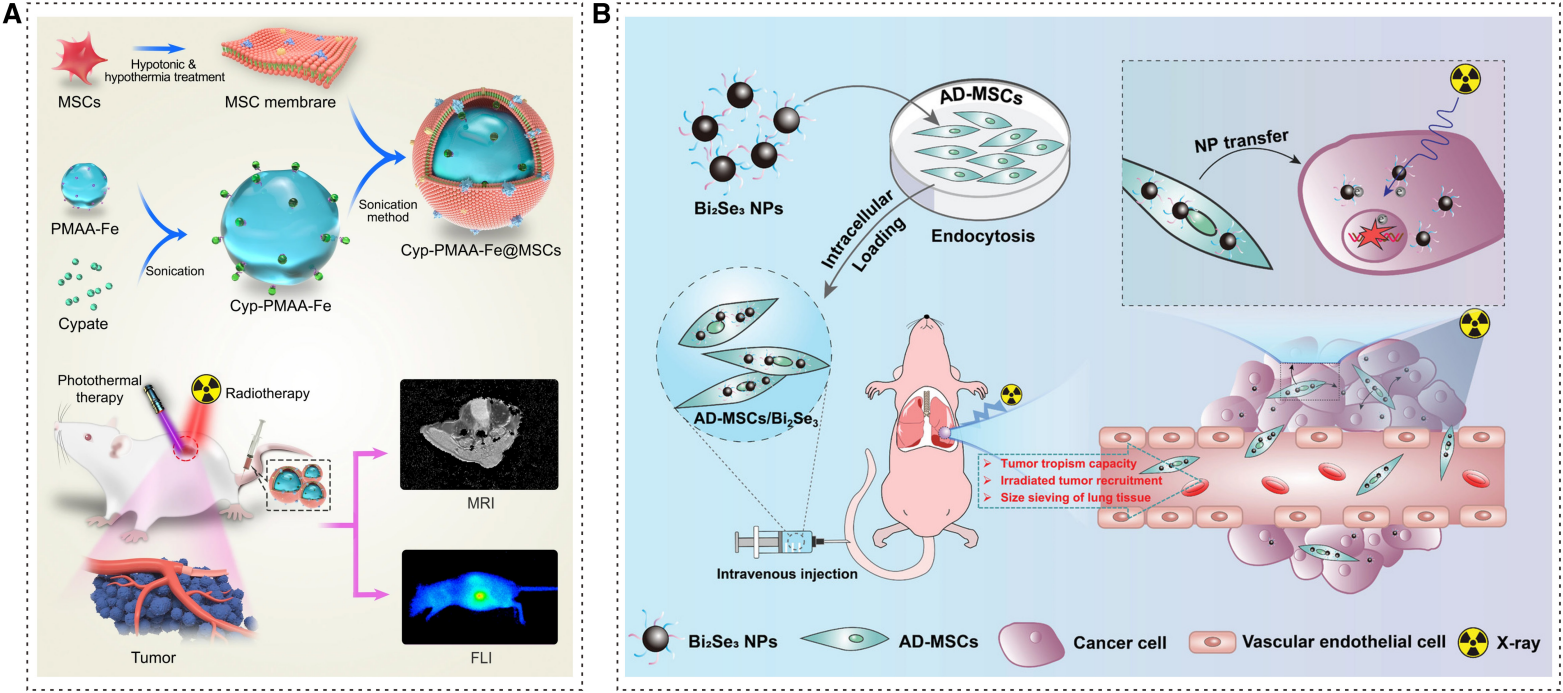
Biomimetic targeting strategies based on a modification of MSCs for RT of NSCLC. (A) Schematic illustration of the construction of Cyp-PMAA-Fe@MSCs nanomedicines for fluorescence imaging and MRI-guided photothermal-enhanced RT of NSCLC. Adapted with permissions from [134]. Copyright 2021, BioMed Central. (B) Schematic diagram of the preparation of AD-MSCs/Bi_2_Se_3_ hybrid. Radiation-sensitive AD-MSCs/Bi_2_Se_3_ are designed to target RT in NSCLC. Adapted with permissions from [136]. Copyright 2022, John Wiley and Sons.

In order to improve the accuracy of NPs targeting cancerous cells, a cell-based drug delivery system using MSCs has been developed and has successfully assisted RT for tumors. It offers several advantages, including the ability to target tumors through chemotaxis and intercellular interactions. Additionally, it has low immunogenicity, which helps the nanomaterial evade immune clearance and surveillance. Moreover, this system allows for long-term retention of the NPs at tumor sites [131]. MSCs have been extensively studied in different areas, including the treatment of NSCLC (Fig. 6) [132,133].

Yin et al*.* developed an MSC-coated nanomaterial with a combination of cypate (Cyp), NP polymethacrylic acid (PMAA), and Fe (III), to construct Cyp-PMAA-Fe@MSCs nanoplatform. PMAA loaded with Fe (III) could be applied to *T*_1_-weighted magnetic resonance imaging (MRI), one of the imaging technologies of radiodiagnosis. Cyp was able to produce fluorescence and had a thermogenic effect under NIR. According to the detection of the fluorescence signal in vivo fluorescence imaging experiments, Cyp-PMAA-Fe@MSCs were proven to have high accumulation in tumor sites. MSC-coated NPs were absorbed by Lewis lung carcinoma (LLC_1_) more easily with no disturbance to the effect of x-rays on LLC_1_ [134]. Moreover, Xiao et al*.* proposed a nanoradiosensitizer delivery system utilizing bismuth selenide (Bi_2_Se_3_), which was induced by adipose-derived mesenchymal stromal cells (AD-MSCs), for targeted RT in NSCLC. AD-MSCs belong to the lineage of MSCs and offer the advantage of easy separation and access [135]. Based on the high x-ray absorption and excellent biocompatibility of Bi, the cytotoxicity of Bi_2_Se_3_ NPs on AD-MSCs was found to be low. Additionally, AD-MSCs were able to effectively maintain the activity of Bi_2_Se_3_ NPs after internalization [48]. In the orthotopic A549 lung tumor-bearing mice model, it is observed that AD-MSCs/Bi_2_Se_3_ can effectively control tumor progression. This could be attributed to their ability to cross physiological barriers and their efficient targeting of tumor centers [136].

### Magnetic targeting strategies for RT of NSCLC

**Fig. 7.**
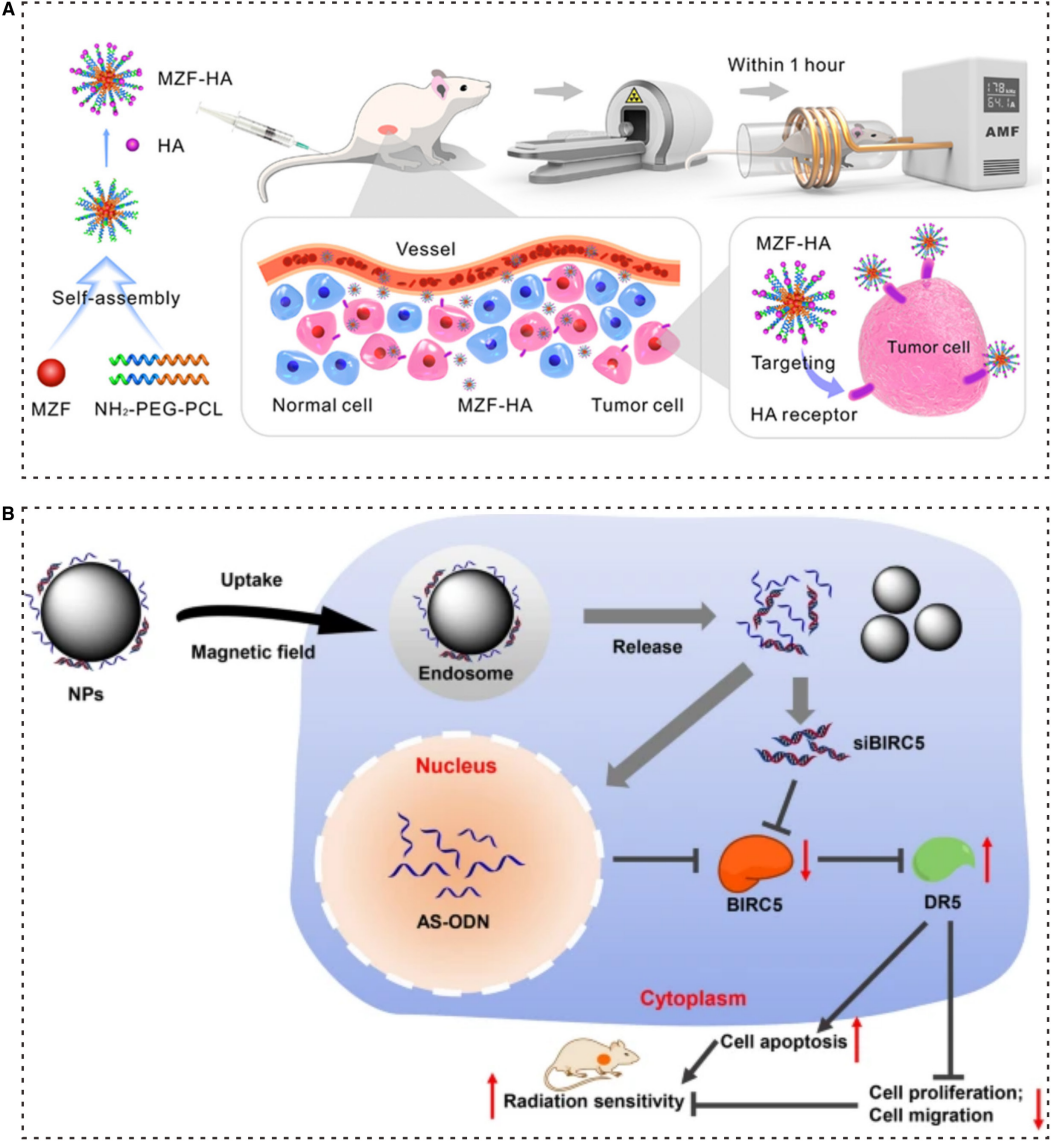
Magnetic targeting strategies for RT of NSCLC. (A) Schematic diagram of MZF-HA-assisted RT-targeted cancer therapy. MZF-HA is enriched in the tumor by binding receptor ligands of HA and CD44, while AMF is applied to gently heat the magnetic NPs to about 43 °C to increase tumor oxygenation, thereby improving the therapeutic effect of RT. Adapted with permissions from [139]. Copyright 2020, American Chemical Society. (B) Fe_3_O_4_ MNPs enhance radiosensitivity through targeted delivery of siBIRC5 and AS-ODN with the assistance of an MF. Adapted with permissions from [142]. Copyright 2021, BioMed Central.

The strategy of using magnetic materials to carry drugs is a promising method to solve the problem of controlling the precise target and release of drugs at tumor sites. Hence, the magnetic drug carrier can be magnetically guided (Fig. 7) [137,138]. Wang et al*.* reported a hyaluronic acid-modified Mn–Zn ferrite magnetic nanoparticles (MZF-HA) used for cancer hyperthermia treatment (HT), which is beneficial to RT of NSCLC. For tumor targets, MZF-HA is highly accumulated in A549 (human lung adenocarcinoma cell line), which expresses CD44 (HA receptor) that can bind to its ligand. Furthermore, under an alternating magnetic field (AMF), MZF can generate thermal energy, which improves the hypoxic transmission electron microscopy (TEM) by increasing tumor oxygenation. They confirmed that MZF-HA can improve the effect of RT treatment by remodeling the TME and targeting tumors efficiently with a combination of RT and HT [139].

Peng et al*.* reported the MFP-FePt-GO NCs, a layered magnetic GO and polyethylene glycol drug delivery system achieving co-delivery of metronidazole and 5-fluorouracil (5-FU), which have an effect on NSCLC for enhancing radiosensitivity. Metronidazole together with IR causes DNA damage, but its high toxicity can damage the surrounding tissues. 5-FU is applied to disturb the synthesis of DNA in lung cancer therapy. The dual delivery system offers a comprehensive treatment of the 2 drugs. Compared with graphene, GO’s surface contains several functional groups that make GO more biocompatible [140]. According to the assay in vitro, MFP-FePt-GO NCs can selectively act on NSCLC cells. As the radiosensitizer, MFP-FePt-GO NCs improve radiosensitivity by leading to mitochondrial dysfunction. Meanwhile, the disorder of mitochondria leads to an increasing level of ROS and the expression of antioxidant proteins, which causes more NSCLC cells to undergo apoptosis. Moreover, this research also suggested that MFP-FePt-GO NCs prevent the repair of DNA damage to help enhance radiosensitivity. Combined with RT, MFP-FePt-GO NCs have the potential to suppress NSCLC cell migration and invasion [117]. Chen et al*.* also reported a co-delivery magnetic targeting strategy with nanotechnology for NSCLC. The study aimed to investigate the effect of Fe_3_O_4_ magnetic nanoparticles (MNPs) on the radiosensitivity of lung adenocarcinoma cells. Fe_3_O_4_-MNPs were confirmed to have the ability of drug targeting and cell regulation [141]. By co-delivering negatively charged small interfering RNA against baculoviral IAP repeat containing 5 (siBIRC5) and antisense oligodeoxynucleotide (AS-ODN), MNPs down-regulated the expression of BIRC5 in lung adenocarcinoma and improve the absorptivity of AS-ODN and small interference RNA (siRNA), which can inhibit gene. As an innovative solution, Fe_3_O_4_ MNPs-AS-ODN/siBIRC5 better enhances the radiosensitivity of lung adenocarcinoma cells [142].

### Nanosensitization based on high-atomic number nanomaterials for RT of NSCLC

**Fig. 8.**
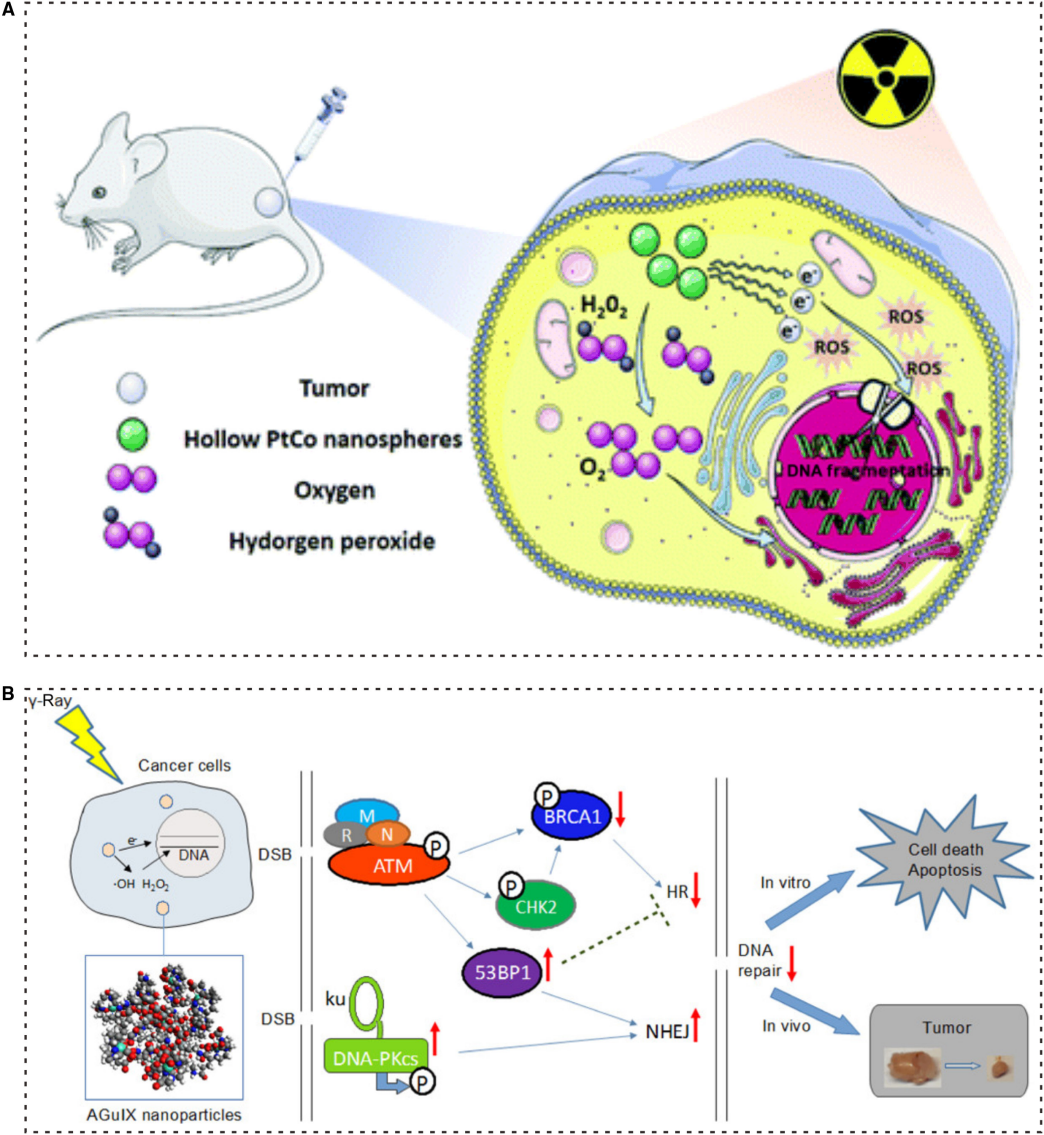
Nanosensitization based on high-atomic number nanomaterials. (A) Mechanism of PtCo NSs enhanced tumor RT. PtCo NS could significantly inhibit tumor growth, simultaneously relieving tumor hypoxia with good biocompatibility and biosafety. Adapted with permissions from [145]. Copyright 2021, Royal Society of Chemistry. (B) High-efficiency gadolinium-based NP AGuIX used in MRI-guided RT and as a potent radiosensitizer to inhibit NSCLC. Adapted with permissions from [149]. Copyright 2020, American Chemical Society.

Extensive experiments have been conducted to verify the effectiveness of high-atomic number nanomaterials in treating cancer, including NSCLC. High-atomic number nanomaterials are constructed with high-atomic number and serve as important radiosensitizers capable of damaging tumors through photoelectric action. In RT, these nanomaterials possess a higher x-ray coefficient than soft tissue. As a result, they can increase the local radiation dose deposition in cells, releasing a significant number of charged particles and radiation photons. This process can directly damage DNA or generate more ROS by dissociating water, thereby enhancing the radiosensitivity of tumor cells. Furthermore, high-atomic number nanomaterials can also be loaded with various chemotherapy, gene therapy, and immunotherapy drugs. This allows for a more precise and systematic therapeutic approach [99,143]. Furthermore, nanostructured metals have demonstrated exceptional radiosensitivity (Fig. 8). Recently, Li et al*.* proposed using hollow PtCo nanospheres (NSs) as radiosensitizers. The experimental results, obtained through the use of a dissolved oxygen meter and intracellular H_2_O_2_ monitoring, demonstrated that PtCo NSs can alleviate tumor hypoxia by converting H_2_O_2_ to O_2_ after irradiation. The researchers also speculated that both Pt and Co elements have the potential to catalyze oxygen doubly. This chemical mechanism plays a role in reducing radioresistance and regulating the hypoxia of the TME. Additionally, due to the high atomic number of nanomaterials, PtCo NSs can cause DNA damage by accumulating in tumor cells at high doses. Furthermore, experiments monitoring the impact of PtCo NSs on NSCLC cells show that PtCo NSs can increase the generation of ROS in these cells. When combined with IR, PtCo NSs can suppress DNA damage repair while elevating the expression of 53BP-1 and γ-H2AX, both of which are proteins associated with DNA damage. In vitro and in vivo assays have confirmed that PtCo NSs enhance the effectiveness of RT [144,145].

Gd chelates play a vital role as contrast agents in MRI, helping with imaging guidance. In addition to their function in MRI, Gd chelates also have important applications as radiosensitizers. A series of studies on gadolinium-intercalated carbon dots (Gd@Cdots) have been conducted. The research showed that Gd@Cdots can accumulate in tumors via EPR, which potentially enables efficient energy utilization [146]. By conducting assays in mice bearing H1299 tumors derived from human NSCLC, Gd@Cdots have shown the potential to inhibit tumors and sensitize RT treatment. In a similar study, Ma et al*.* used positron emission tomography (PET) to study NP retention in vivo and selected pPD-Gd@C-dots as an effective radiosensitizer due to their persistence in tumors, compared to CA-Gd@C-dots. Gd@Cdots carried carboxylic acid (CA-Gd@C-dots) and an amino group (pPD-Gd@C-dots) [76].

AGuIX NPs, which consist of gadolinium ions (Gd^3+^) encapsulated within polysiloxane NPs, have the potential as radiosensitizers [147]. A recent study has confirmed the radiosensitizing effect of AGuIX NPs in the treatment of cancer while also uncovering their potential mechanism of promoting ferroptosis through modulation of the NRF2-GPX4 signaling pathway [148]. Concurrently, related research has demonstrated the application of AGuIX NPs in NSCLC [149]. Cells exhibited arrest at the G_2_-M phase, suggesting that AGuIX may impair the cells’ proliferative and DNA repair capabilities. The NPs also affected the homologous recombination repair (HRR) pathway, which is a principal mechanism for repairing DNA double-strand breaks (DSBs) in tumor cells. By diminishing the activity of key HRR proteins such as RAD51 and the phosphorylation levels of BRCA1, AGuIX NPs attenuated the cells’ ability to repair DNA damage. As a novel radiosensitizing agent, AGuIX NPs are already undergoing clinical trials, which establishes a solid foundation for their application in the RT of NSCLC [150].

However, NPs with a high atomic number face several challenges, including heavy metal toxicity, large size, and a low rate of cell uptake. To address these concerns, Lee et al*.* conducted a study on Gd@Cdots that have a high gadolinium content and an ultrasmall size. The combination of gadolinium’s photoelectric effect and the carbon catalyst surface enables the generation of more hydroxyl radicals when exposed to x-rays. Additionally, the ultrasmall size promotes increased cell uptake. Since carbon is biologically and chemically inert, the Gd@Cdots can reduce the toxicity and leakage of gadolinium. Assays conducted by the researchers demonstrated that Gd@Cdots have the ability to enhance the generation of ROS and improve radiosensitivity. Moreover, the particle’s photoelectric and surface effects depend on both of its components, which can be regulated. Therefore, by controlling the structure of the particle, the radiosensitization impact can be amplified [151].

## Conclusion and Prospects

Lung cancer is a condition characterized by a malignant tumor that has a high morbidity and mortality rate, and it often presents in the advanced stage. It is crucial for patients affected by lung cancer to be diagnosed and treated early. NSCLC is a significant type of lung cancer that is considered to be less responsive to RT. Therefore, it is imperative to enhance the effectiveness of therapy with advanced technology. RT is the preferred treatment for almost all stages of NSCLC, so improving precision is equally important. One strategy to optimize RT involves the use of nanomaterials. In this review, we will summarize the various mechanisms by which nanomaterials assist in RT, including their role as nanoradiosensitizers, their ability to assist in remodeling the TME, and their use as nano-based radioprotectors (Table). Importantly, in the field of ITM reconstruction, nano-assisted RT primarily acts through the following mechanisms: (a) ITM reconstruction: This approach aims to reverse immunosuppression, attract a group of immunoreactive cells, and modify the ITM to create a more favorable environment for an immune response. (b) Material properties: The nanomaterials used possess tunable characteristics and are biocompatible, ensuring their proper functioning within the tumor’s context. Additionally, these nanomaterials have the ability to modify the tumor’s acidic and hypoxic microenvironment, thereby increasing the tumor’s sensitivity to RT. (c) Combination therapy: By combining nanotechnology with RT, a synergistic effect can be achieved, known as the abscopal effect. This effect not only inhibits the growth of irradiated tumors but also effectively suppresses the progression of nonirradiated, distant tumors. Specifically, we have discussed the application of nanomaterials in improving the accuracy of RT in NSCLC. This includes biomimetic targeting, magnetic targeting, and nanosensitization using high-atomic number nanomaterials. Moreover, it is noteworthy that combining RT with other technologies, such as immunotherapy, big data and machine learning, and innovative technology in RT like real-time adaptive MRI-based RT, has proven beneficial for NSCLC patients. The growing body of research supports the multifaceted applications of nanomaterials in NSCLC and highlights the potential of nano-assisted RT to significantly improve cancer treatment [4,152,153].

**Table.** Overview of nanotechnology for enhancing the therapeutic effect of RT

Name	Composition	Application	Reference
^125^I-HSA nanoparticles	HSA, ^125^I	Enhance uptake and retention of nanoparticles upon x-rays	[75]
Alb-GNPs	Alb, GNPs	Promote radiosensitivity and the targeted therapy	[84]
Acid-triggered aggregation GNPs	GNPs	Generate more ROS and prolong the retention time in the tumor site	[87]
Au@SA-QBA	AuNPs, SA, QBA	Inhibit oxidative stress to normalize the abnormal tumor vascular microenvironment	[102]
AIM NPs	4PI, CaCO_3_	Modulate the pH and immunosuppressive metabolism of the TME	[106]
αPD-L1@MnO_2_	MnO_2_, αPD-L1	Amplify the cGAS–STING pathway to promote the maturation of DCs	[109]
Bi-nMOF	Bi	Facilitate ICD and diminish the levels of TGF-β	[109]
Smac-TLR7/8 hydrogel	TLR7/8 agonists, Smac N7 peptide	Modulate the polarization of TAMs	[113]
MFP-FePt-GO NCs	MP-GO NCs, FePt NPs	Activate mitochondrial-induced apoptosis and impair DNA damage repair	[117]
PVP-Bi_2_Se_3_@Sec NPs	PVP, Sec, Bi_2_Se_3_ NPs	Enhance radiosensitivity and the immune system	[119]
Cyp-PMAA-Fe@MSCs	Cyp, PMAA nanoparticles, Fe (III), MSC	Allow for multiple bioimaging-guided and photothermal-enhanced RT	[134]
AD-MSCs/Bi_2_Se_3_	Bi_2_Se_3_ NPs, AD-MSCs	Maintain the tumor tropism of Bi_2_Se_3_ nanoradiosensitizer and enhance the effect of RT	[136]
MZF-HA	MZF, HA	Remold the TME and target tumors efficiently	[139]
Fe_3_O_4_-MNPs	Fe_3_O_4_	Co-delivery of siBIRC5 and AS-ODN to enhance radiosensitivity	[142]
PtCo NS	Pt, Co	Improve the generation of oxygen and alleviate tumor hypoxia	[145]
Gd@Cdots	pPD, Gd (NO_3_)_3_	Amplify the radiosensitization impact and reduce heavy metal toxicity	[151]
AGuIX	Gd^3+^	Attenuate the ability to repair DNA damage by diminishing the activity of HRR proteins	[149]

HSA, human serum albumin; ^125^I, radioactive iodine-125; Alb, albumin; GNPs, gold nanoparticles; AuNPs, gold nanoparticles; SA, sodium alginate; QBA, 8-quinoline boric acid; AIM NPs, acidity-IDO1 modulation nanoparticles; αPD-L1@MnO_2_, MnO_2_ anti-programmed death ligand 1 (αPD-L1); Bi-nMOF, Bi-based nanoscale metal-organic framework (nMOF); Smac-TLR7/8 hydrogel, Toll-like receptor 7/8 (TLR7/8) agonists Smac N7 peptide; MP-GO NCs, mixture of PEGylated GO nanocomposites; FePt NPs, FePt nanoparticles; PVP, poly(vinylpyrollidone); Sec, selenocysteine; Bi_2_Se_3_ NPs, bismuth selenide nanoparticles; Cyp, cypate; PMAA nanoparticles, polymethacrylic acid nanoparticles; MSC, mesenchymal stem cell; AD-MSCs, adipose-derived mesenchymal stromal cells; MZF, Mn–Zn ferrite magnetic nanoparticles; HA, hyaluronic acid; Fe_3_O_4_-MNPs, Fe_3_O_4_ magnetic nanoparticles; siBIRC5, small interfering RNA against baculoviral IAP repeat containing 5; AS-ODN, antisense oligodeoxynucleotide; Pt, platinum(V); Co, the intermediates of the Co–B–O complex; pPD, *p*-phenylenediamine; Gd (NO_3_)_3_, gadolinium nitrate hexahydrate; Gd@Cdots were synthesized by hydrothermal reaction; AGuIX, polysiloxane nanoparticles containing gadolinium ions (Gd^3+^).

While the development of nano-assist technologies holds promise for optimizing RT outcomes, there are several challenges that need to be addressed. First, it is crucial to tackle RT resistance and effectively manage the dynamic changes in the TME. Although nanomaterials can reverse immunosuppression, they may also disrupt the immune balance of the organism, potentially leading to more severe inflammation and immune responses. In vitro experiments cannot accurately replicate the in vivo immune status due to the complexity of the immune system. Consequently, this poses significant challenges in understanding the metabolism and immunity of TME, as well as the clinical application of nanomaterials. In addition, it is essential to fully consider patient characteristics when developing a targeted and effective RT plan. This presents a significant challenge for both radiation therapists and clinical practitioners.

The current clinical application of nanomedicines still faces several common difficulties, which has led to many works remaining in the preclinical research stage. First, the cost-effectiveness of nanotechnology in clinical settings is a major challenge. The raw materials and production equipment required for many nanomedicines are expensive, making it difficult for developing countries and regions to afford them. Additionally, the complex synthesis steps and strict storage conditions needed for many nanotherapy strategies pose challenges in ensuring the stability of nanomedicines. Moreover, the clinical application of nanomedicines necessitates rigorous clinical experimental verification and safety evaluation, which is often beyond the financial capabilities of most medical institutions and research units.

Consequently, due to economic constraints, most research teams still primarily focus on basic research, which remains far from achieving real clinical transformation [24]. The issue of biosafety is another important aspect that nanomedicines cannot overlook. While numerous studies have demonstrated the biocompatibility of nanomedicines in vivo at various levels, including their toxicity to cells and preclinical animal models, doubts remain about whether the results from these experiments accurately reflect the effects of nanomaterials on the human body. The complex physical and chemical properties of nanomaterials may lead to unforeseen harm to the body, even while they are intended to provide therapeutic benefits or stimulate immune responses. To bridge this gap, comprehensive and in-depth research is still needed to better understand the toxicity and intricate interactions between nanomaterials and human cells [23,154].

In summary, nanomedicine is an innovative approach to precision RT and has made significant progress in recent years. In addition to optimizing and exploring nanomaterials, there is a central challenge of improving our understanding of cancer pathogenesis in different tissues and gaining deeper insights into therapeutic mechanisms. As awareness of nanomedicine continues to increase, there will be a surge in more comprehensive and in-depth research on the use of adjuvant nanomedicine for treating NSCLC. The promising prospect is that strategies for nano-assisted RT may transition from preclinical trials to practical clinical applications, ultimately enhancing cancer treatment.

## Ethical Approval

The authors declare that human or animal ethics approval was not needed for this study.
